# Transvaginal hemiperitoneal cervical cerclage for cervical insufficiency: A single‐center retrospective study

**DOI:** 10.1002/ijgo.70520

**Published:** 2025-09-17

**Authors:** Xin Zhao, Meicheng Wang, Le Cai, Yan Sun, Yansong Liu

**Affiliations:** ^1^ Shenyang Women's and Children's Hospital Shenyang Liaoning Province China

**Keywords:** cervical cerclage, cervical insufficiency, McDonald, Shirodkar, transvaginal hemiperitoneal

## Abstract

**Background:**

Cervical insufficiency (CI) is thought to be responsible for 8% of miscarriages and preterm births. Cervical cerclage is the main treatment for CI. There are different approaches to cervical cerclage, and it is particularly important to find a method of cervical cerclage that is simple to perform and results in better pregnancy outcomes.

**Objective:**

This study investigates the clinical efficacy of transvaginal hemiperitoneal cervical cerclage in the treatment of CI and its impact on maternal and fetal outcomes.

**Methods:**

We performed a descriptive retrospective single‐center study, including all patients who had a transvaginal hemiperitoneal cervical cerclage between January 1, 2020, and December 31, 2023. The main outcomes measured were pregnancy outcomes.

**Results:**

Twenty transvaginal hemiperitoneal cervical cerclages were performed over the period studied: 35% had a history of cervical conization, 65% had history of one or more mid‐trimester miscarriages, and 15% had a history of a failed emergency or prophylactic cerclage. The median gestational age (GA) at cerclage placement was 14.19 ± 2.88 weeks of gestation (WG). There was one case of delivery between 28 and 32 weeks, and there were five cases of delivery between 32 and 37 weeks and 11 cases of delivery over 37 weeks, of which three cases were in pregnancy, at gestational weeks of 16, 18, and 21 weeks, respectively. The mean gestational age of cerclage removal, was 36.42 ± 2.76 (WG).

**Conclusion:**

The transvaginal hemiperitoneal cervical cerclage is simple to operate, not limited by gestational age, and less invasive and has a low cost. It is effective in patients with a short cervix and has good pregnancy outcomes.

## 1 INTRODUCTION

Cervical insufficiency (CI) is defined as the painless dilatation of the cervix in the second trimester or the early third trimester (<34 weeks), which leads to late abortion or preterm birth. The diagnosis of CI is essentially based on the analysis of obstetric history. A history of two or more mid‐trimester miscarriages and/or preterm deliveries following “silent” cervical dilatation without uterine contractions nor chorioamnionitis nor preterm prelabor rupture of membranes (PPROM) is highly suggestive of CI.

The incidence rate is approximately 0.1%–2%^1^, ^2^ and accounts for 15% of recurrent spontaneous abortions between the 16th and 28th weeks.^3^ CI is associated with 8% of miscarriages and preterm births in the second and third trimesters of pregnancy.^4^ Pregnant women with CI are prone to recurrent miscarriages in the second and third trimesters of pregnancy, resulting in adverse pregnancy outcomes.

Cervical cerclage technique has been used for more than 50 years in the prevention of pre‐term births associated with cervical incompetence, is the main treatment for CI, which can avoid cervical dilation, reduce the risk of infection caused by vaginal pathogens ascending through the cervix to the uterine cavity, and prolong the gestational age.^5^, ^6^, ^7^ Depending on the surgical route, cervical cerclage can be divided into transvaginal cervical cerclage (TVCC), laparoscopic abdominal cervical cerclage (LACC), and transabdominal cervical cerclage (TACC). At present, the most widely used TVCC procedures in clinical practice include McDonald^8^ and Shirodkar,^9^ and the TACC procedure is mainly uterine isthmus cerclage.^10^ Shirodkar TVCC requires an incision of the cervix–vaginal mucosa and separation of the bladder–cervical space and rectovaginal space. The suture is located above the main ligament, the ligation position is high, and the operation is complex,^9^ which requires high surgical skills and cannot be widely used. McDonald TVCC uses cerclage to perform a simple purse suture directly at the junction of the cervix and vagina, without separating the bladder and rectum and at a lower ligation position than the Shirodkar procedure, but the method is simple and easy to perform.^8^ For women with CI who have previously failed TVCC, LACC can ensure that the cerclage position is at the level of the internal cervical ostium, which is a better pregnancy outcome than TVCC.^11^, ^12^ However, the disadvantage is the need for cesarean section to terminate the pregnancy. In addition, if an unexpected situation occurs during pregnancy, laparoscopic or even open circular removal or cesarean section is required, increasing the risk of surgical trauma in pregnant women.^10^

Therefore, it is particularly important to find a method of cervical cerclage that is simple to perform and results in better pregnancy outcomes. This study assesses the use of the transvaginal hemiperitoneal cervical cerclage technique and analyzes the pregnancy outcomes and related factors to provide a new method and clinical basis for the treatment of pregnant women with CI.

## 2 MATERIALS AND METHODS

### 2.1 Study design

This was a retrospective, descriptive single‐center study conducted at Shenyang Women's and Children's Hospital. The study retrospectively analyzed clinical data of patients who underwent transvaginal hemiperitoneal cervical cerclage between January 1, 2020, and December 31, 2023.

### 2.2 Study setting

This study was conducted at Shenyang Women's and Children's Hospital, a tertiary women's and children's hospital with an annual volume of over 6000 obstetric surgeries, including approximately 100–120 cervical cerclage procedures yearly. This study was approved by the Research Ethics Committee of the hospital (PJ‐SC‐2024‐012). Prior to enrollment, participants were provided with detailed information regarding the study's objectives and methodology, and written informed consent was obtained from all individuals. Data were collected after obtaining written informed consent.

### 2.3 Participants

A diagnosis of cervical insufficiency can be accompanied by one or more of the following factors: (1) previous McDonald's circumcision has failed; (2) the cervix is extremely short (<25 mm) and there is no suitable suture position in the vaginal area of the cervix; and (3) a history of conization of the cervix leads to a shortening of the cervical structure.

### 2.4 Intervention

Diagnostic criteria for cervical insufficiency: (1) ≥2 no abortion signs, painless late miscarriages, or history of preterm birth and (2) <2 no abortion signs and painless late miscarriages or preterm births, accompanied by one of the following conditions: (i) transvaginal ultrasound measurement of cervical length (CL) ≤2.5 cm before 24 weeks of gestation, accompanied by progressive cervical dilation and shortening of the cervical canal; (ii) non‐pregnant transvaginal ultrasound measurement CL ≤2.5 cm; and (iii) the No. 8 cervical dilation rod in the non‐pregnant period passes through the internal cervical opening without resistance.

*Inclusion criteria*: (1) previous McDonald's circumcision has failed; (2) the cervix is extremely short (<25 mm), and there is no suitable suture position in the vaginal area of the cervix; and (3) a history of conization of the cervix leads to a shortening of the cervical structure.

*Exclusion criteria*: (1) multiple pregnancies; (2) termination of pregnancy unrelated to CI after cerclage, such as termination of pregnancy due to fetal abnormalities, pregnancy complications or complications of pregnant women, and abnormalities of fetal appendages; and (3) active bleeding and infection.

During the study period, a total of 420 cervical cerclage procedures were performed at our center. Among them, 20 patients were selected for transvaginal hemiperitoneal cervical cerclage based on the following specific criteria: (1) previous failure of McDonald cerclage; (2) extremely short cervix (<25 mm) with no suitable suture position in the cervicovaginal region; and (3) history of cervical conization resulting in shortened cervical structure. Patients eligible for conventional McDonald or Shirodkar procedures were excluded from this study.

Before surgery, all patients provided written informed consent for transvaginal hemiperitoneal cervical cerclage and informed consent met the requirements of the hospital ethics committee. All the operations were performed by gynecologic surgeons with extensive experience in transvaginal surgical techniques (>500 major gynecologic transvaginal surgery procedures per surgeon). Data extracted from the medical records included age, body mass index, parity, CL, causes of cervical cerclage, preoperative infection indicators, operation time, intraoperative bleeding, cerclage gestational age, postoperative tocolytic drug application, hospital stay, distance between the cerclage and the internal cervical ostium, CL during delivery, surgical complications, complications during childbirth, pregnancy outcome, and other indicators.

### 2.5 Surgical techniques: Transvaginal hemiperitoneal cervical cerclage

**FIGURE 1:**
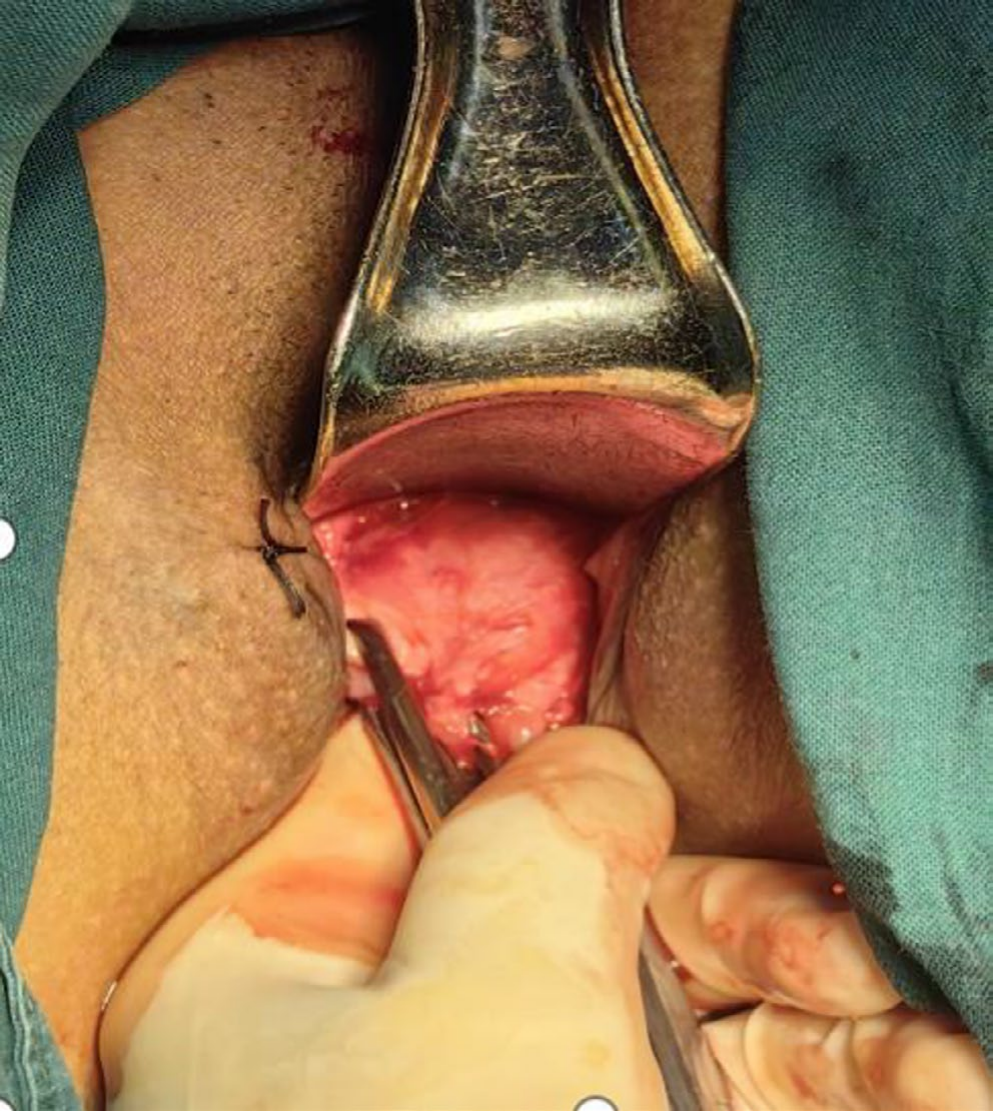
The cervical isthmus was exposed.

**FIGURE 2:**
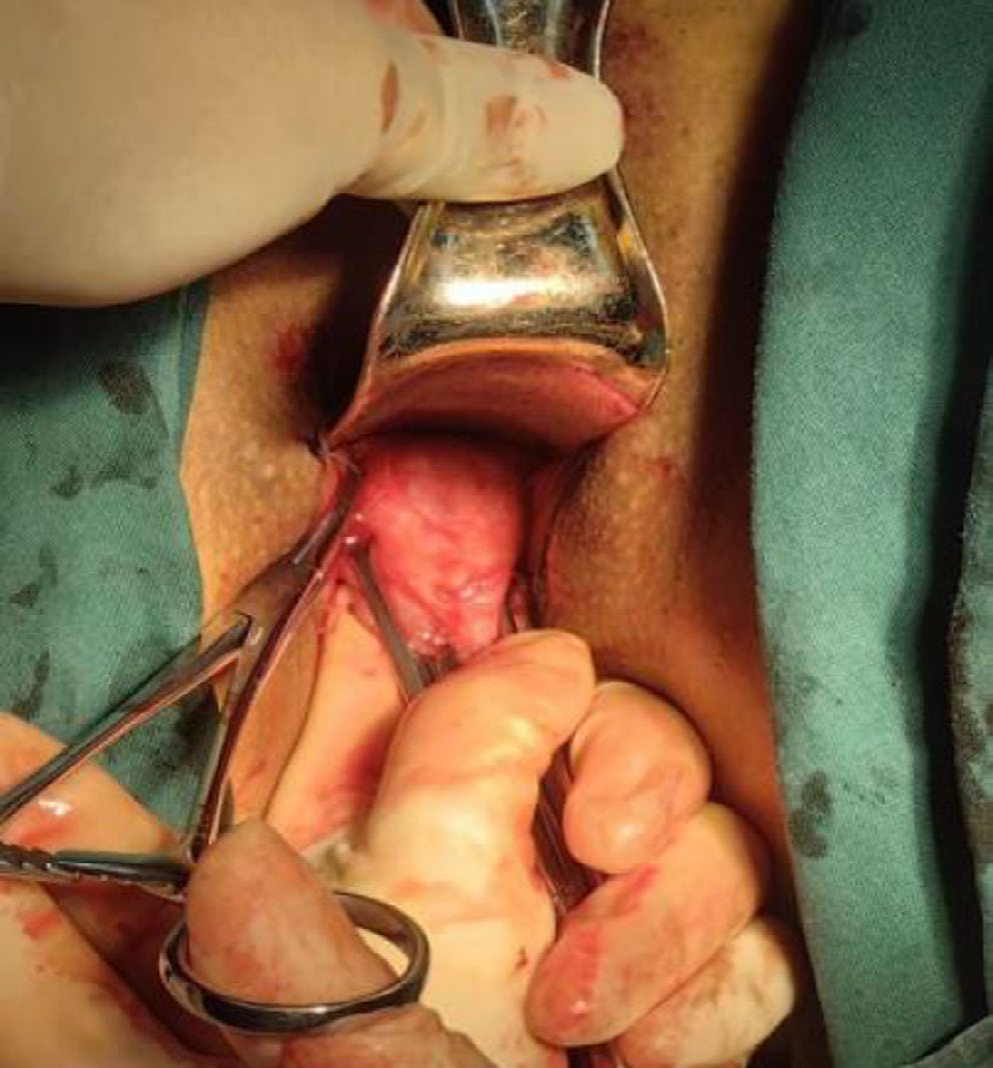
Puncture point of the Mersilene polypropylene tape (Right side).

**FIGURE 3:**
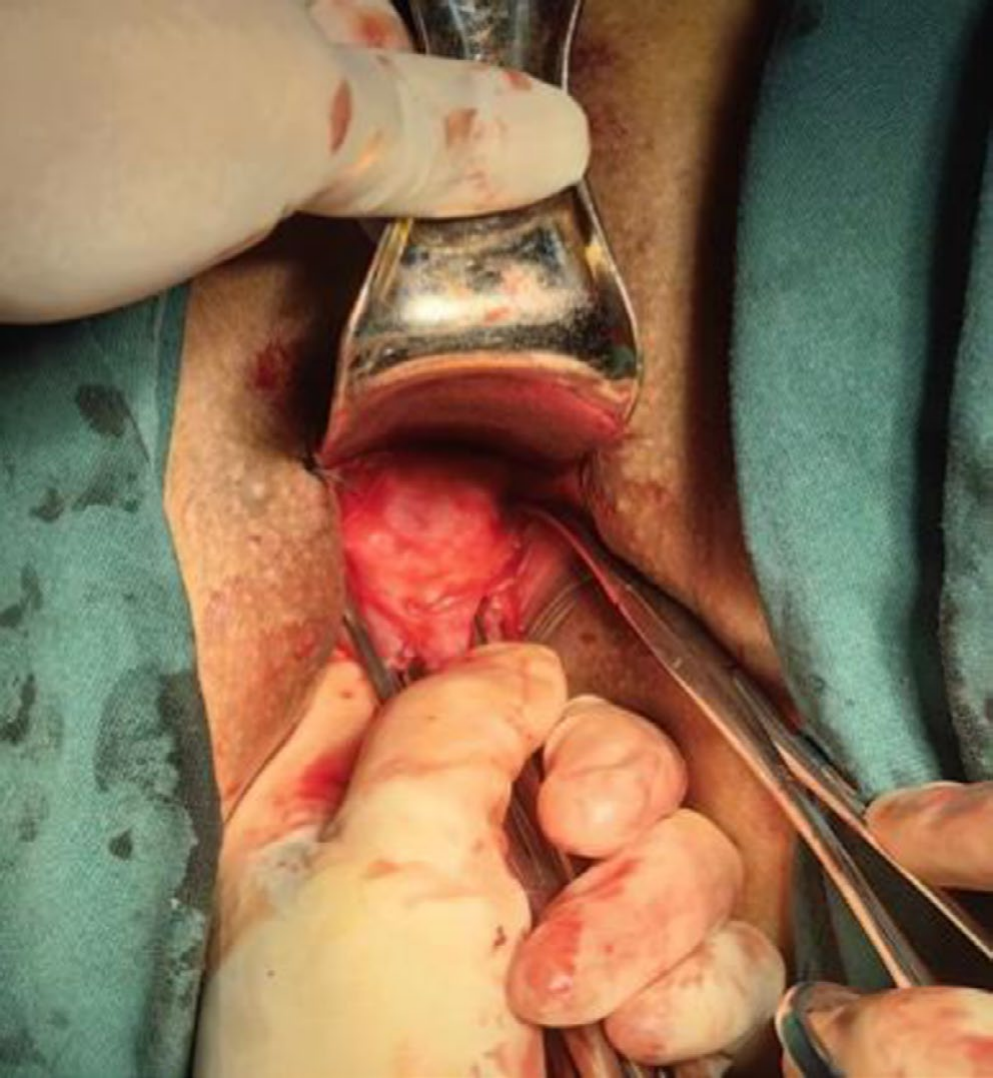
Puncture point of the Mersilene polypropylene tape (Left side).

**FIGURE 4:**
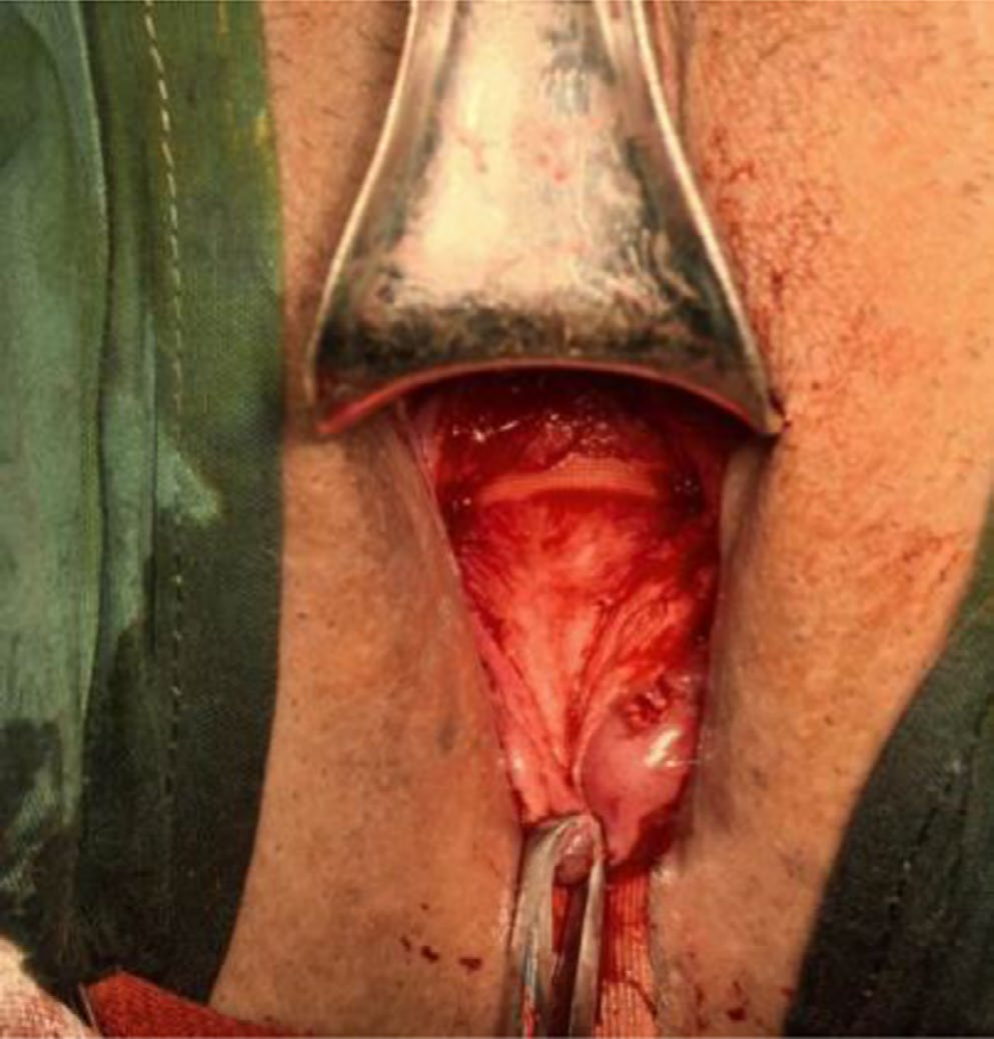
The suture band is spread flat on the surface of the cervix.

**FIGURE 5:**
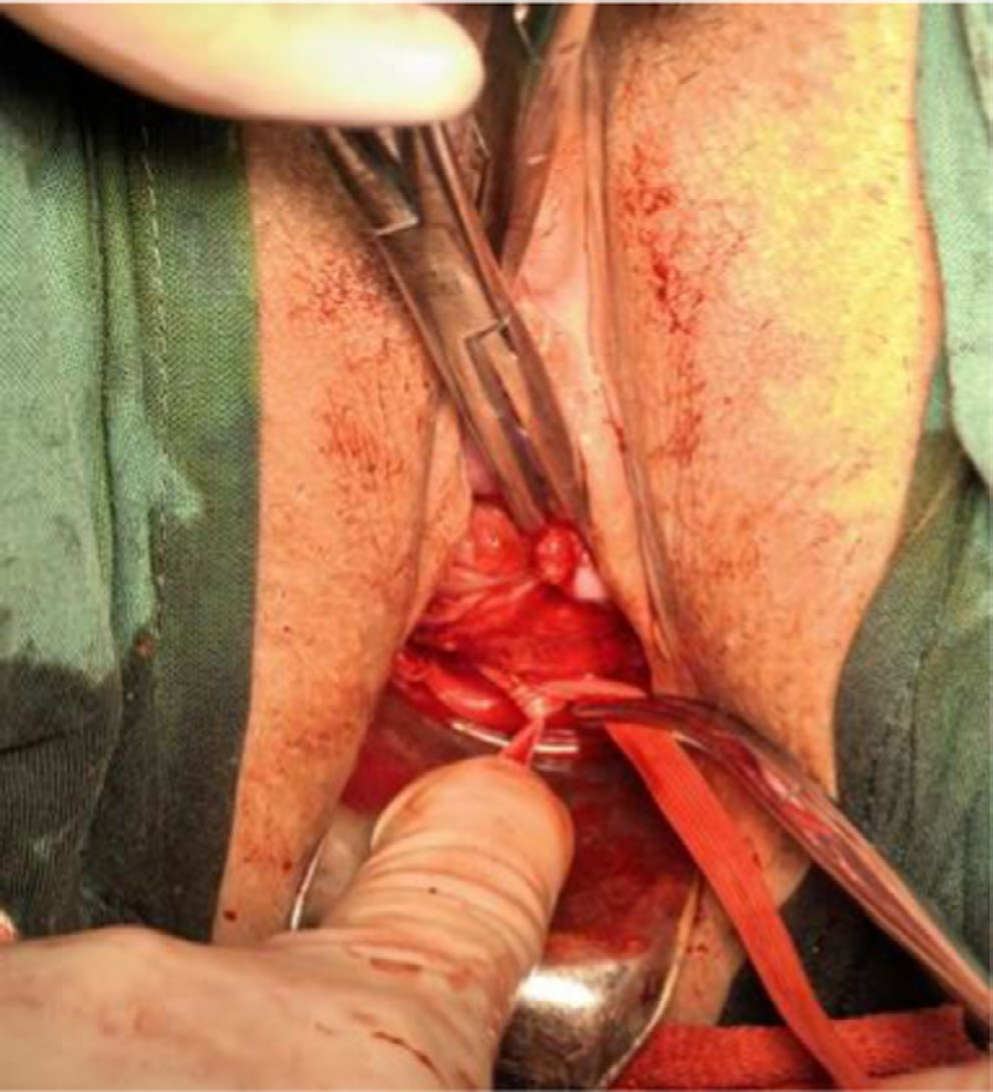
Tie three knots in the space behind the cervix.

Continuous epidural block anesthesia is delivered with 1.5–1.8 mL of 0.5% bupivacaine to the subarachnoid space until the sensory plane reaches T10. The pregnant woman takes the bladder lithotomy position and keeps their head low and their hips high. Following routine disinfection and urinary catheter, use vaginal retractors (upper and lower lobe hooks) to expose the cervix. Disinfect the cervix and vagina again. Using Allis clamps (10 cm), clamp the vagina on both sides of the anterior lip of the cervix, gently pulling downward, opening the anterior vaginal fornix 2–3 cm, pushing the bladder upwards until the cervical isthmus is exposed (Figure 1). Allis clamp the mucosa of the posterior vaginal wall, opening the posterior fornix 2 cm. Separating the uterorectal space, the surgeon's right hand palpates the pelvic peritoneum at the beginning of the sacral ligament obliquely from the posterior fornix space, and the left index finger strokes to the side of the cervix in the anterior separated space. Until the fingers of the right hand are touched, the corresponding place between the two fingers is the circular belt puncture point, located at the junction of the sacral ligament origin and pelvic peritoneum, close to the outer side of the cervical isthmus. The left index finger is enclosed in an anti‐puncture glove, and the peritoneum at the beginning of the sacral ligament is pushed up using Mersilene 5 mm polypropylene tape (Mersilene, Ethicon, manufactured by Johnson & Johnson). A puncture needle enters the needle from the puncture point determined in front of the cervix (Figures 2 and 3). It crosses the peritoneum from the anterior direction and backwards, and punctures it from the vaginal–rectal space where the left index finger rises; that is, the semi‐peritoneal puncture is more conducive to the fixation of the circular band, and the opposite side is treated in the same way. The suture band is spread flat on the surface of the cervix (Figure 4), with the distance between the cerclage and internal cervical ostium controlled within 0–0.8 cm (verified by transvaginal ultrasound postoperatively). Tie three knots in the space behind the cervix (Figure 5). Ensuring there is no active bleeding, stuff a piece of PVP gauze in the vagina, remove the anal protective towel, and the operation is completed. The semi‐peritoneal puncture refers to the technique of passing the suture through the peritoneal reflection at the origin of the sacral ligament without fully entering the peritoneal cavity, ensuring secure fixation while avoiding intraperitoneal organ injury.

### 2.6 Perioperative management

All women received intravenous prophylactic antibiotics before the vaginal incision, and the antibiotics were continued until 24 h after surgery if the temperature is normal. On postoperative day 1, the urinary catheter and the vaginal tamponade were removed. Transvaginal ultrasound was performed 3 days after surgery to evaluate the position of the band, such as during pregnancy to evaluate the intrauterine condition of the fetus. Three days after the operation, patients were discharged. If a patient who underwent cerclage before pregnancy needs to use contraception for one month after the operation, then they can try to get pregnant.

### 2.7 Postoperative follow‐up

Postoperative follow‐up included, first, fortnightly visits from 14 to 28 weeks of gestation, then monthly visits until delivery. Second, transvaginal ultrasound was performed using GE Voluson E10 at each visit to measure CL. CL was measured from internal to external os in the midsagittal view. Criteria for hospitalization included cervical shortening >50% from baseline or symptoms of threatened preterm birth (uterine contractions and vaginal discharge). Third, follow‐up was conducted by obstetricians specializing in high‐risk pregnancies, with midwives performing weekly home visits for stable patients after tocolysis.

The cerclage is removed at 37–38 WG. Transvaginal surgery is performed in the operating room under spinal anesthesia or light sedation. During labor before 37–38 WG, the cerclage is removed in the delivery room under epidural anesthesia.

### 2.8 Data collection

Patient demographics, obstetric and gynecological histories, as well as the indication for cerclage, were preoperatively recorded. The gestational age of pregnancy at the time of cerclage was recorded. Perioperative data included the duration of the procedure, time in hospital, blood loss, and surgical complications. Obstetric outcomes were collected directly from obstetricians and pediatricians providing care, hospital charts, and telephone interviews with patients. Data were checked by a gynecologist from Shenyang Women's and Children's Hospital during the period of data collection so that missing data and data with logical errors could be identified and corrected in time.

### 2.9 Study outcomes

The main outcomes measured were pregnancy outcomes. We considered that the transvaginal hemiperitoneal cervical cerclage had failed if there was a delivery <28 WG, and success was defined as a viable livebirth at 28 WG or more. Preterm birth categories were very preterm (28–31 + 6 weeks), moderate preterm (32–33 + 6 weeks), and late preterm (34–36 + 6 weeks). Surgical complications were reviewed, including infection (postoperative fever >38.5°C lasting >24 h with positive bacterial culture), significant bleeding (>50 mL intraoperative or postoperative), bladder/ureteral injury, and rectal injury.

### 2.10 Statistical analysis

Statistical procedures were performed using the SPSS software version 23.0. In cases of a large amount of missing data, the deletion method or the substitution method was selected. The data in this study were basically complete. Innovative surgical methods were carried out, and the adverse pregnancy outcomes after the operation were significantly improved. The application scope is gradually expanding, but the current audience is still a small group of people, and the sample size is relatively small. However, the measurement data of the enrolled population were tested using the K‐S method, which conformed to the normal distribution. Meanwhile, the 95% confidence interval of the measurement data was increased to reduce the effect of the lack of sample data. Counting data is calculated using IQR%.

## 3 RESULTS

Between January 1, 2020, and December 31, 2023, 420 cerclage procedures were performed in the center, of which 20 were transvaginal hemiperitoneal cervical cerclage.

### 3.1 Maternal characteristics

The median age was 33.35 ± 4.77 years, and the median body mass index was 26.66 ± 5.83 kg/m^2^. Fourteen patients (16.4%) had a history of gynecological surgery, including seven cervical conizations, one hysteroscopic adhesion separation, one hysteroscopic electroresection of residual pregnancy tissue, three hysteroscopic electroresection of endometrial polyps, and two laparoscopic resections of ovarian cysts.

The mean gravity was 3 and the mean parity was 0. There were two nulliparas (10%). Sixty‐five percent of the patients had a history of one or more mid‐trimester miscarriages.

In three cases, a cerclage had been performed in the previous pregnancy, including two cases of McDonald's emergency cerclage aborted at 26 and 27 weeks. One case was transabdominal cerclage, and labor was induced at 20 weeks of pregnancy due to “fetal malformation,” and the cerclage was removed. Only six patients did not have a history of mid‐trimester miscarriage and/or preterm delivery. The reason for performing cerclage in these patients was a very short cervix (Table 1).

**TABLE 1:** Medical and socio‐demographic characteristics of patients with transvaginal hemiperitoneal cervical cerclage.

Variables	Study cohort, mean ± SD/*n*	95%CI/*n*%/IQR%
Age, years	33.35 ± 4.77	31.12–35.58
Body mass index, kg/m^2^	26.66 ± 5.83	23.93–29.39
Profession		
Manager or self‐employed	6 (6/20)	30
Employee	12 (12/20)	60
Unemployed	2 (2/20)	10
Smoking	2 (2/20)	10
History of gynecological surgery		
Conization of cervix	7 (7/20)	35
Hysteroscopic adhesion separation	1 (1/20)	5
Hysteroscopic electroresection of residual pregnancy tissue	1 (1/20)	5
Hysteroscopic electroresection of endometrial polyps	3 (3/20)	15
Laparoscopic resection of ovarian cysts	2 (2/20)	10
Gravity		
1	4 (4/20)	3
2	4 (4/20)	
3	8 (8/20)	
4	2 (2/20)	
6	1 (1/20)	
7	1 (1/20)	
Parity		
0	16 (16/20)	0
1	4 (4/20)	
History of cerclage		
History of failure of McDonald	2 (2/20)	10
History of failure of emergency McDonald	2 (2/20)	10
History of miscarriage		
History of mid‐trimester miscarriage	13 (13/20)	65
History of threatened PTL	1 (1/20)	5
History of PPROM	10 (10/20)	50
Additional evidence of cervical insufficiency	0	0

Abbreviations: BMI, body mass index; PPROM, preterm prelabor rupture of membranes; PTL, preterm labor.

### 3.2 Cerclage characteristics

The mean gestational age at cerclage was 14.19 ± 2.88 (WG). Indications for cerclages were three cases of previous cervical cerclage failure, two cases of CL <25 mm, 10 cases of historical cause of cerclage, and five cases of previous preterm birth and miscarriage due to non‐CI, but the patient had a history of cervical conization, which was not in accordance with the guidelines for cerclage. Eight out of 20 patients had postoperative intravenous infusion of magnesium sulfate, and 12 out of 20 had postoperative intravenous infusion of magnesium sulfate plus oral progesterone. Magnesium sulfate, 1 g/h maintenance for tocolysis; tocolysis is stopped if no signs of preterm labor are observed for ≥24 h. Oral progesterone (200 mg daily) should be administered if there is a history of preterm birth. All the modified Shirodkar procedures were prophylactic. There were five cases of natural conception and 15 cases of in vitro fertilization‐assisted conception.

The preoperative leukocytes were 9.68 ± 1.85 (109/L) and C‐reactive proteins were 3.25 ± 1.08 (mg/L). The mean CL before the cerclage was 27.32 ± 0.75 mm, the operation time was 27.05 ± 7.01 min, the intraoperative bleeding was 12.10 ± 6.98 mL, and there were no intraoperative and postoperative complications. Ultrasound shows that the cerclage is located at the internal cervical orifice, accounting for 16/20, and 4/20 ultrasound indicates that the cerclage is 0.5–0.8 cm horizontal from the internal cervical ostium (Table 2).

**TABLE 2:** Pregnancy characteristics of patients with transvaginal hemiperitoneal cervical cerclage and intra‐ and postoperative details.

Characteristics	Study cohort, mean ± SD/*n*	95%CI/*n*%/IQR%
Pregnancy		
Spontaneous pregnancy	5 (5/20)	25
IVF	15 (15/20)	75
Gestational age at cerclage	14.19 ± 2.88	12.82–15.57
Cerclage indications		
Failure of previous cerclage	3 (3/20)	15
Extremely short cervix (< 25 mm)	2 (2/20)	10
History cause	10 (10/20)	50
History of previous preterm birth and miscarriage not attributable to cervical insufficiency	5 (5/20)	25
Preoperative indicators		
WBC (10^9^/L)	9.68 ± 1.85	8.81–10.55
CRP (mg/L)	3.25 ± 1.08	3.03–4.94
Vaginal microbiology positive	0	0
Operation time (min)	27.05 ± 7.01	23.77–30.33
Intraoperative bleeding (mL)	12.10 ± 6.98	8.83–15.37
Complications during surgery	0	0
Hospitalization after cerclage (day)	5.73 ± 1.42	4.77–6.68
Post‐surgery complications	0	0
Cervical length before cerclage (mm)	27.32 ± 0.75	24.69–30.31

Abbreviations: CRP, C‐reactive protein; WBC, white blood cell.

### 3.3 Pregnancy outcomes

The mean gestational age of cerclage removal was 36.42 ± 2.76 WG. The cerclage removals were performed mostly in the operating room. Five of the 17 patients did not have the cerclage removed; they had their pregnancies terminated by cesarean section, and the patients and their families had a need for a second child and requested that the cerclage be retained.

There was one case of delivery between 28 and 32 weeks. There were five cases of delivery between 32 and 37 weeks and 11 cases of delivery over 37 weeks, of which three cases were in pregnancy, gestational weeks of 16, 18, and 21 weeks respectively. There were six cases of vaginal delivery, 10 cases of elective cesarean section, and one case of emergency cesarean section, the fetus was in intrauterine distress with Apgar score of 10. The newborn weighed 3130.91 ± 883.02 g. Most newborns (12) had no complications. Five were transferred to the neonatal intensive care department for prematurity under 36 weeks of gestation, two for suspicion of infection, two for jaundice requiring phototherapy, and one for growth restriction (Table 3).

**TABLE 3:** The situation of the cerclage and pregnancy outcomes of patients with transvaginal hemiperitoneal cervical cerclage.

Characteristics	Study cohort, mean ± SD/*n*	95%CI/*n*%/IQR%
Ultrasound indicates the position of the cerclage		
The level of the internal cervical ostium	16 (16/20)	80
From the internal cervical ostium		
< 0.5 cm	2 (2/20)	10
From theinternal cervical ostium		
> 0.5 < 0.8 cm	2 (2/20)	10
Cerclage removal		
Yes	12 (12/17)	70.59
No	5 (5/17)	29.41
Cerclage removal gestational age (median, WG)	36.42 ± 2.76	35.64–37.22
Cervical length before cerclage removal	3.35 ± 0.62	3.21–3.49
Gestational age at delivery (median WG)	37.38 ± 1.03	36.06–38.70
< 28 WG	0	0
28−31 + 6 WG	1 (1/17)	5.88
59	2 (2/17)	11.76
64	3 (3/17)	17.65
≥ 37 WG	11 (11/17)	64.71
Delivery mode		
Spontaneous vaginal delivery	6 (6/17)	35.29
Instrumental delivery	0	0
Planned C‐section	10 (10/17)	58.82
Emergency C‐section	1 (1/17)	5.88
Complications of childbirth	0	0
Neonatal survival rate	17/17	100
Newborn weight (kg)	3130.91 ± 883.02	2744.85–3516.68

Abbreviation: WG, weeks of gestation.

Long‐term follow‐up data (median 12 months postpartum) showed no maternal complications such as cervical stenosis or pelvic adhesions. Neonatal follow‐up up 6 months of age revealed no neurodevelopmental abnormalities in 16/17 cases; one preterm infant had mild developmental delay requiring early intervention.

## 4 DISCUSSION

In this study, 90.91% of patients with second‐trimester miscarriage or preterm birth underwent transvaginal hemiperitoneal cervical cerclage, and the average number of weeks of termination was 37.38 ± 1.03 weeks. This is consistent with the data reported in the previous literature. In a retrospective study by Joy Bloomfield et al., 55 patients underwent Shirodkar cerclage; 74.5% had history of one or more mid‐trimester miscarriages. The main outcomes measured were delivery at or beyond 24 WG. The perinatal success rate of Shirodkar cerclage was over 90%.^9^

The median gestational age at birth was higher in our study compared to other studies.^13^ The reason might be that the reasons for cerclage are different. In some literature, it is mainly used for emergency cervical cerclage, and the included researchers themselves have a high rate of preterm birth, and the object of our study is mainly to conduct preventive cervical cerclage, so this may be that the number of weeks we terminate the pregnancy is larger.

Several studies have compared CL before and after prophylactic or therapeutic cerclage, and some have demonstrated that CL might increase after cerclage.^14^, ^15^, ^16^ In our study, the CL before cerclage was 2.73 ± 0.75 cm and the post‐cerclage CL was 3.35 ± 0.62 cm. This is consistent with the results of the above study.

According to the anatomical and physiological characteristics of the cervicovaginal vault, the posterior fornix is 1–2 cm deeper than the anterior fornix, and the anterior and posterior fornix is incised during the operation. The bladder is pushed up, and the internal cervical orifice is exposed, so that the cerclage is on the same plane of the anterior and posterior fornix, parallel to the levator ani plate, so that the whole plane is evenly stressed, which has the advantage of vaginal cerclage and also of optimizing the focus point of the cervix in the pelvic floor. It is more effective for patients with a history of previous cervical conization, short cervicovaginal region, and proximal cervical abduction. In this group, both prophylactic cervical cerclage and therapeutic cervical cerclage were administered, and satisfactory pregnancy outcomes were obtained. The transvaginal hemiperitoneal cervical cerclage ensures a higher position of cerclage at the internal cervical ostium, reinforcing the weight‐bearing tension of the cervix and partially sealing the external cervical ostium.

There is evidence that higher placement of the cervical stitch is a key to success.^17^ In a review published by Berghella,^18^ it was stated that the residual CL after cerclage should be greater than 2 cm to be effective in the prevention of pre‐term birth; therefore, the cerclage suture must be applied as close as possible to the internal cervical os. The McDonald procedure is, thus, limited in the height that can be attained, while the Shirodkar technique allows for higher placement and, in comparison with the McDonald procedure, there was a greater contribution to residual cervical height. There was also better support of the cervical tissue.^19^ Transvaginal hemiperitoneal cervical cerclage could be a good alternative because it allows an attempt at vaginal birth and its success rate is comparable.

The Shirodkar cerclage is more invasive than the McDonald cerclage because it requires dissection to be inserted higher on the cervix, but it is less invasive than transabdominal techniques by laparotomy or laparoscopy, requiring delivery by cesarean section, which is not devoid of maternal risks.^20^ Even though laparoscopic cervical cerclage has been reported to remove the cerclage vaginally, it is an isolated case. Burger et al.^21^ reported three cases of removing transvaginal cerclage stitches in the second trimester. Because the cerclage is located in the peritoneum, the circumferential cavity can only be exposed after the circumflex peritoneum is opened and the circumflex peritoneum is removed, which increases the probability of secondary injuries such as to the bladder and intestinal tract, and the operation is difficult. Transvaginal hemiperitoneal cervical cerclage does not require the opening of the bladder and rectal reflexion peritoneum and is less invasive than laparoscopic patients. The comparative advantage of the transvaginal hemiperitoneal cervical cerclage is to allow for vaginal delivery.

We have several cases of patients who have undergone cesarean section to terminate their pregnancies, and the cerclage is not removed at the same time during the operation. Similar to the patients after laparoscopic cerclage, the cerlap is retained during cesarean section, and the next pregnancy still has a role, but due to the relatively short follow‐up time of our cases, after transvaginal hemiperitoneal cervical cerclage, understanding whether there will be long‐term complications in patients who terminate the pregnancy by cesarean section without removal will require long‐term follow‐up.

Based on our study, we can draw several conclusions. Advantages of surgery include, first, that transvaginal surgery is performed, with less trauma and no incision in the abdomen, with an aesthetically satisfactory result. Second, for patients with a history of cervical conization, short cervicovaginal part, and near‐flat cervix, it is difficult to operate using McDonald surgery due to the limited position of cervical cerclage suture. Further, this surgical cerclage can reach the position near the internal cervical ostium, especially for patients with a history of cervical conization surgery and other cervical shortening. Third, intraperitoneal puncture on the lateral side of the cervix is more conducive to the fixation of the cerclage and avoids the displacement of sutures. The cerclage knot is not exposed to the vagina to avoid infection. Fourth, the cerclage knot is not exposed to the vagina to avoid infection. Fifth, laparoscopic cerclage can be performed at 8–14 weeks of gestation; for those at more than 14 weeks of gestation, the uterus is enlarged, resulting in no operation space to perform the laparoscopy. If a laparotomy is performed, the trauma can be considerable. This surgery is performed vaginally, and the surgical operation can be performed at any gestational week during pregnancy. Sixth, it can also be used for emergency cervical cerclage. Seventh, this procedure can be performed before or after pregnancy. Eighth, it is convenient to remove the cerclage through the vagina, and the cerclage can be delivered vaginally after removing the cerclage, which avoids the need for cesarean section of the laparoscopic cerclage or the need to induce labor due to abnormal fetal development during pregnancy and the need to remove the cervical cerclage through the abdominal approach after laparoscopic cerclage, which reduces the secondary surgical trauma of pregnant women. Finally, the cerclage is placed at a higher position of the internal cervical orifice, which strengthens the tension of the cervix bearing weight, assists the internal cervical opening to bear the gravity of the fetus and fetal appendages in the late stage of pregnancy, provides a certain degree of support for the weakened cervical structure, and maintains the length of the cervix.

There are also limitations of transvaginal hemiperitoneal cervical cerclage. First, to reach a higher position near the cervical internal orifice, the surgeon needs to be very familiar with pelvic floor anatomy, have rich experience in transvaginal surgery, and have a good sense of touch. They need to touch the pelvic peritoneum at the beginning of the sacral ligament and puncture and suture the peritoneum at the beginning of the sacral ligament. For those who are not skilled in vaginal surgery, a certain amount of technical practice is required to ensure the safety and effectiveness of the surgery. Second, there were four patients with non‐contraction‐like pain in the lower abdomen after surgery, which was considered to be related to cervical traction. Third, when removing the cerclage vaginally, it is often necessary to remove it under anesthesia.

This study has several strengths. First, the computerized medical records gave access to over 4 years of data, with very little missing information. Second, all procedures were performed by the same medical team. Third, transvaginal surgery is familiar to most gynecologists, and the method is easy to learn and master.

However, this study has some limitations that should be considered. First, this was a single‐center, retrospective study. Selection bias is inevitable due to the retrospective design, as patients were selected based on specific clinical indications (previous failure of other cerclage techniques or short cervix), which might limit generalizability. Second, this study includes a small number of patients. The small sample size reduces the statistical power. However, 80 additional cases have been accumulated since 2024, and a prospective study is underway to validate these findings. Third, there was no control group for comparison. The absence of a control group prevents direct comparison with other cerclage techniques, and future studies should include historical controls matched by indication. Fourth, there is no rigorous trial protocol for postoperative pregnancy prenatal examination, and some patients return to local hospitals for prenatal examination. The obstetric level is inconsistent, which might lead to biased pregnancy outcomes. Fifth, not all women will benefit in the same way. Pregnancy outcomes depend on baseline characteristics, and cervical cerclage might have different outcomes depending on the indication. Sixth, follow‐up duration is relatively short, and long‐term maternal outcomes (e.g., cervical function in subsequent pregnancies) require further investigation. Seven, we need prospective and multicenter studies to clarify whether there will be complications such as cervical incision and infection in individual patients who have not removed the cerclage during the operation and whether the cerclage can be retained during cesarean section, like for laparoscopic cerclage, and whether the cerclage can still be effective during cesarean section.

## 5 CONCLUSION

Transvaginal hemiperitoneal cervical cerclage has the advantages of higher cerclage placement than a McDonald cerclage, compared to laparoscopic cerclage, while allowing for vaginal delivery. The transvaginal hemiperitoneal cervical cerclage is simple to operate, not limited by gestational age, and less invasive and less expensive. It is effective in patients with a short cervix and has good pregnancy outcomes.

## AUTHOR CONTRIBUTIONS

XZ was involved in the conception and design of the study and drafted and performed the statistical analysis, XZ, LC, and YS interpreted the data, XZ and WM wrote the manuscript; YS designed this operation. YS was in charge of the operation. All authors reviewed the results. All authors contributed to the article and approved the submitted version.

## FUNDING INFORMATION

Shenyang Science and Technology Bureau plan project: Study on the feasibility of transvaginal hemiperitoneal cervical ceration in the treatment of cervical insufficiency.

## CONFLICT OF INTEREST STATEMENT

The authors declare that the research was conducted in the absence of any commercial or financial relations that could be construed as a potential conflict of interest.

## DECLARATIONS

This study was approved by the Ethical Committee of Shenyang Women's and Children's Hospital. Written informed consent was obtained from the patients for the publication of any potentially identifiable images or data included in this article. Written informed consent was obtained from all participants.

## Data Availability

The original contributions presented in the study are included in the article/supplementary materials, and further inquiries can be directed to the corresponding author. All methods were performed in accordance with relevant guidelines and regulations.
